# 

**Published:** 1862-05

**Authors:** 


### CONTENTS.

ORIGINAL ESSAYS AND COMMUNICATIONS.

Arsenic—Its Application and Use. By Dr. WM. A. PEASE........ 197
Metals and Alloys. By Dr. B. WOOD,........ 210
Constitutional Causes of Caries. By H. H. BLACK,........ 216
Success in the Dental Profession. By C. C. DILLS, D. D. S........ 218

SELECTIONS.

Dentistry as a Profession,........ 221
Dental Hospitals,........ 225
New Method of Giving Chloroform........ 228
Syphillitic Malformation of Teeth,........ 229
Concerning the Opening of Abscesses,........ 230
Improved Water Apparatus for Lathe,........ 233

EDITORIAL.

Rubber Attachment,........ 234
American Dental Association,........ 235
Dentistry as a Profession,........ 236
Dental Convention,........ 237

NOTICES.

The American Dental Convention,........ 238
The American Dental Association,........ 239

OBITUARY

James Robinson, Esq.,........ 239

